# Subunit contribution to NMDA receptor hypofunction and redox sensitivity of hippocampal synaptic transmission during aging

**DOI:** 10.18632/aging.102108

**Published:** 2019-07-24

**Authors:** Ashok Kumar, Jeffrey S. Thinschmidt, Thomas C. Foster

**Affiliations:** 1 Department of Neuroscience, McKnight Brain Institute, University of Florida, Gainesville, FL 32611, USA; 2 Department of Pharmacology and Therapeutics, University of Florida, Gainesville, FL 32611, USA; 3 Genetics and Genomics Program, University of Florida, Gainesville, FL 32611, USA

**Keywords:** aging, hippocampus, CA1 pyramidal neurons, NMDA receptor current, redox state, dithiothreitol

## Abstract

We examined the contribution of N-methyl-D-aspartate receptor (NMDAR) subunits in the redox-mediated decline in NMDAR function during aging. GluN2A and GluN2B selective antagonists decreased peak NMDAR currents to a similar extent in young and aged animals, indicating that a shift in diheteromeric GluN2 subunits does not underlie the age-related decrease in the NMDAR synaptic function. Application of dithiothreitol (DTT) in aged animals, increased peak NMDAR synaptic currents, prolonged the decay time, and increased the sensitivity of the synaptic response to the GluN2B antagonist, ifenprodil, indicating that DTT increased the contribution of GluN2B subunits to the synaptic response. The DTT-mediated increase in NMDAR function was inhibited by partial blockade of NMDARs, and this inhibition was rescued by increasing Ca^2+^ concentration in the recording medium. The results indicate that DTT-mediated potentiation requires Ca^2+^ influx through NMDAR activity. Finally, redox regulation of NMDAR function depends on the activity of Ca^2+^/calmodulin-dependent protein kinase II (CaMKII). The results indicate that activity-dependent NMDAR synaptic plasticity is suppressed by redox-mediated inhibition of CaMKII activation during aging. The redox regulation of NMDARs represents a suppression of a metaplasticity mechanism, which can disrupt synaptic plasticity and cognition associated with neurological or psychiatric diseases, and aging.

## Introduction

The function of N-methyl-D-aspartate receptors (NMDARs) have a profound influence on synaptic plasticity, cognition, psychiatric diseases, and the connectivity of neural networks [1,2]. For example, redox regulation of NMDAR function during development influences the formation of synaptic connections and neuronal circuits involved in schizophrenia [3,4]. In adults, a redox-mediated NMDAR hypofunction results in depressive-like behavior [5]. With advancing age, metabolic redox stress induces NMDAR hypofunction, weakening synaptic plasticity, and impairing cognition [6–10].

Redox regulation of NMDAR function can be studied by examining the effects of oxidizing or reducing agents on the NMDAR component of synaptic transmission. In aged animals, the reducing agent, dithiothreitol (DTT), increases the NMDAR synaptic response and rescues synaptic plasticity [6–9,11–13]. In contrast, application of oxidizing agents decreases NMDAR responses and impairs the induction of synaptic plasticity, specifically in young animals [6,14]. The results point to a redox sensitive mechanism in mediating the well-characterized decrease in the CA3-CA1 NMDAR synaptic response of older-memory impaired animals, and suggests that redox regulation of NMDARs influences synaptic plasticity during aging [15–18].

The exact mechanism for redox regulation of NMDARs during aging is unclear, but likely involves thiol S-nitrosylation of cysteine residues or formation of disulfide bonds between cysteine residues of NMDAR subunits or proteins involved in NMDAR regulatory processes [19,20]. NMDARs are heterotetramers and previous research has focused on diheteromeric NMDARs with two GluN1 subunits and two identical GluN2 subunits, either GluN2A or GluN2B. The diheteromeric GluN2 subunits have different kinetics and are differentially sensitive to Zn^2+^ and redox reagents. For example, the GluN1 and GluN2A subunits have extracellular cysteine residues, and under oxidizing conditions, S-nitrosylation or disulfide bond formation of cysteine residues decreases receptor function [21–24]. Over the course of development, many brain regions exhibit an increase in the decay rate of NMDAR synaptic responses resulting from an increased contribution of GluN2A to NMDAR responses [25,26]. Due to the redox sensitive cysteine residues of GluN2A, a shift in the ratio of GluN2A/GluN2B could render older synapses more susceptible to redox regulation. In this case, DTT should increase the GluN2A contribution to the synaptic response.

In addition, an intracellular oxidized redox state is predicted to impair signaling involved in regulating NMDAR function and receptor trafficking. The DTT-mediated increase in the NMDAR response is blocked by inhibition of Ca^2+^/calmodulin-dependent protein kinase II (CaMKII) [6]. In turn, CaMKII regulates NMDAR trafficking to the synapse [27,28] and CaMKII increases the contribution of GluN2B to the synaptic response [28–32]. If redox regulation is acting through NMDAR plasticity involving GluN2B, DTT application should increase the GluN2B contribution to the synaptic response.

The current study, recorded synaptically evoked excitatory postsynaptic currents (EPSCs) from CA1 hippocampal pyramidal neurons and field excitatory postsynaptic potentials (fEPSPs) from CA3-CA1 synapses, and examined the contribution of GluN2A and GluN2B subunits to the decline in NMDAR synaptic function during aging, and the DTT-induced enhancement of NMDAR-mediated synaptic transmission. The results indicate that the age-related decrease in the NMDAR response is not due to a shift in the ratio of diheteromeric GluN2A/GluN2B subunits at the synapse. Furthermore, the DTT-mediated increase in the synaptically evoked NMDAR current involves an increase contribution of GluN2B. The redox regulation of the NMDAR response was dependent on the level of NMDAR activity and kinase activation. Together, the results suggest that increased oxidative stress during aging suppresses NMDAR activity-dependent plasticity.

## RESULTS

### Decreased NMDAR synaptic currents during aging

**Figure 1:**
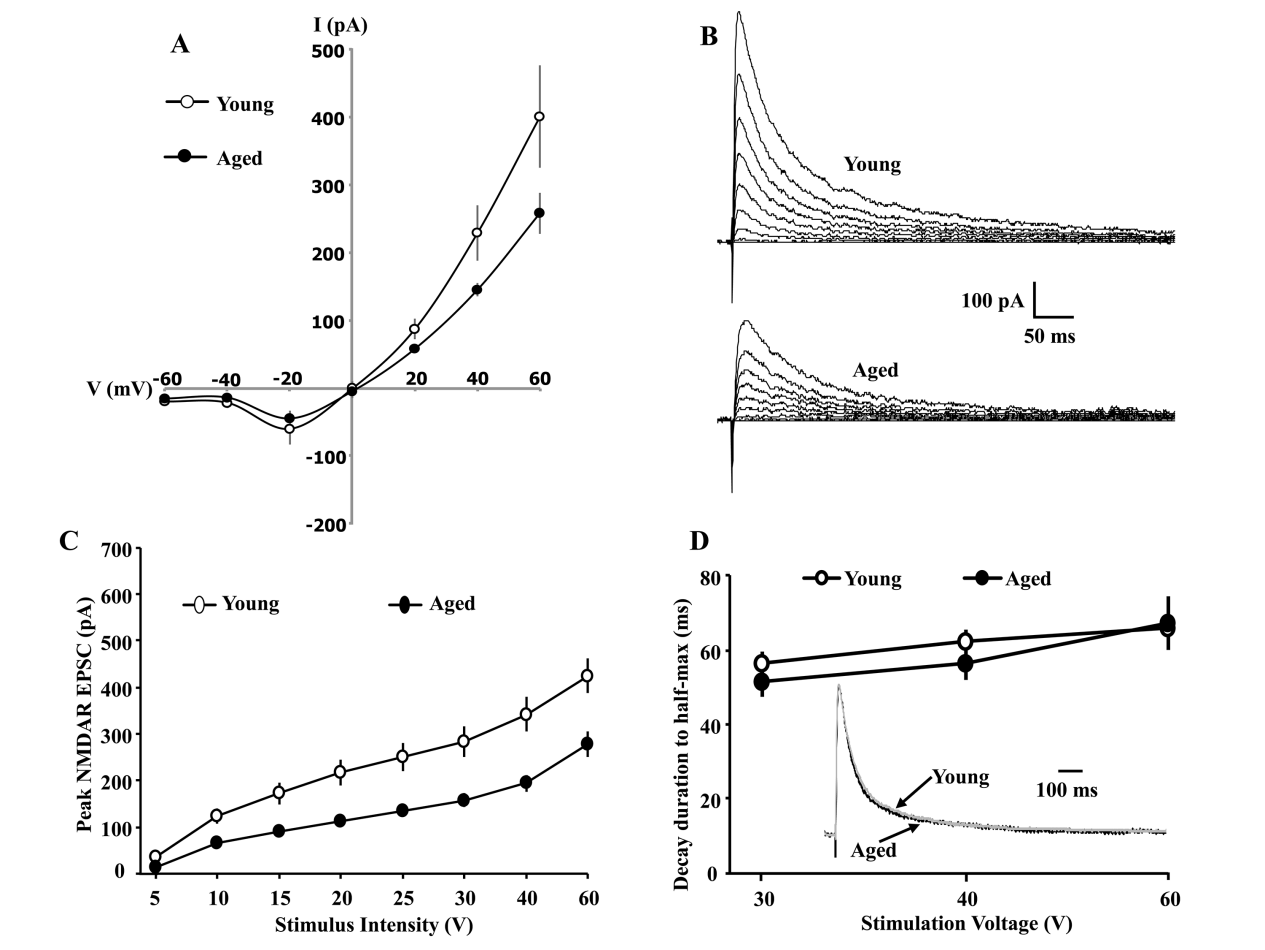
**Whole-cell patch clamp recording from CA1 hippocampal pyramidal neurons of aged and young animals demonstrating the current-voltage relationship and synaptic decay duration**. (**A**) The current-voltage relationship was recorded from CA1 pyramidal neurons from young (11/4 cells/animals) and aged (9/4 cells/animals) animals. When cells are clamped at positive voltages, the currents are outward and larger currents are observed for young animals. The reversal potential is near 0 mV for both age groups. When cells are clamped at negative voltages, currents are inward and reduced, consistent with Mg^2+^ blockade of the NMDAR channel. Examination of peak amplitude and time to half-decay of the NMDAR EPSC during aging. The cells were voltage clamped at +40 mV. (**B**) Representative traces evoked by the eight different stimulation intensities and recorded from young (top) and aged animals (bottom). (**C**) A decrease in the peak NMDAR EPSC was observed across the range of stimulation intensities for CA1 pyramidal cells recorded from aged animals (filled circle, n = 26/14 cells/animals), relative to cells from young animals (open circle, n = 20/9 cells/animals). (**D**) The mean (±SEM) time for the EPSC to decay to 50% of the peak for the three highest stimulation intensities. The inset shows the time course of the EPSC, evoked by 40 V stimulation, across all CA1 pyramidal cells recorded from young (gray trace, n = 20/9 cells/animals) and aged (dark trace, n = 26/14 cells/animals) animals. For each cell, the response amplitude evoked by 40 V stimulation was normalized to the peak of the response.

Whole-cell patch-clamp recordings of synaptically evoked NMDAR-mediated EPSCs were obtained from CA1 pyramidal cells of hippocampal slices obtained from young (11/4 cells/animals) and aged (9/4 cells/animals) animals. For a subset of cells, isolation of NMDA currents was confirmed by bath application of AP-5 (100 µM) (Suppl. Fig. 1). The EPSCs were recorded at holding voltages between -60 and +60 mV in 20 mV steps. No age-related difference was observed for intrinsic properties, including access resistance, membrane resistance, and capacitance (Table 1). For negative voltages, currents were inward with reduced amplitudes, consistent with Mg^2+^ blockade of the NMDAR channel. The reversal potential was calculated from a regression of responses through -20, 0, and +20 mV. The reversal potential of the synaptically evoked current was near 0 mV, consistent with NMDAR permeability to multiple cations, and no age-related difference was observed for the reversal potential (young: -6.9 ± 3.1 mV; aged: -3.4 ± 3.2 mV). A repeated measures Analysis of variance (ANOVA) on EPSC amplitudes across the voltage steps indicated a tendency (p = 0.051) for an interaction of age and membrane potential due to an increase in outward currents for young animals at positive potentials (Fig. 1 A, Suppl. Fig. 2 A & B).

**Table 1:** Intrinsic properties of CA1 hippocampal pyramidal neurons.

**Cell Properties**	**Young Control**	**Aged Control**	**Young DTT**	**Aged DTT**
**Access resistance (MΩ)**	33.94 ± 2.3	32.82 ± 2.5	27.51 ± 3.1	33.41 ± 7.9
**Membrane resistance (MΩ)**	159.29 ± 7.5	147.77 ± 6.9	152.28 ± 14.7	138.37 ± 7.0
**Capacitance (pF)**	141.58 ± 5.7	140.14 ± 8.7	141.58 ± 5.7	158.54 ± 16.6

Intrinsic properties of CA1 hippocampal pyramidal neurons recorded by whole-cell patch clamp from young control (n = 25/11 cells/animals), young DTT (n = 8/2 cells/animals), aged (n = 24/14, cells/animals), and aged DTT (n = 7/3 cells/animals) animals.

To examine synaptic input-output relationships, cells were held at +40 mV and NMDAR EPSCs were recorded across a range of stimulation intensities (5, 10, 15, 20, 25, 30, 40, and 60 V). For each stimulation intensity, the peak amplitude of the EPSC was obtained from CA1 cells of aged (n = 24/14 cells/animals) and young (n = 25/11 cells/animals) animals. A repeated measures ANOVA across stimulation intensities indicated an interaction of age and stimulation intensity [F(7,329) = 6.58, p< 0.0001] and the age difference was due to larger peak EPSC in young animals (Fig. 1 B, C). The results confirm that NMDAR synaptic responses are reduced during aging [15–18].

### The GluN2A/GluN2B subunit composition is not altered with advanced age

The subunit composition can be studied by examining the time course of NMDAR synaptic responses. An increase in the ratio of GluN2A/GluN2B subunits results in a more rapid decay, producing a shortened synaptic response [25,33]. To examine age differences in the decay of the synaptic response, the EPSC responses for 30, 40, and 60 V stimulation were normalized to the peak of the response, and the time to decay to 50% was calculated. A small, nonsignificant, decrease in the decay rate was observed for aged animals, suggesting that aging is not associated with a large shift in the GluN2A/ GluN2B ratio (Fig. 1 D).

**Figure 2:**
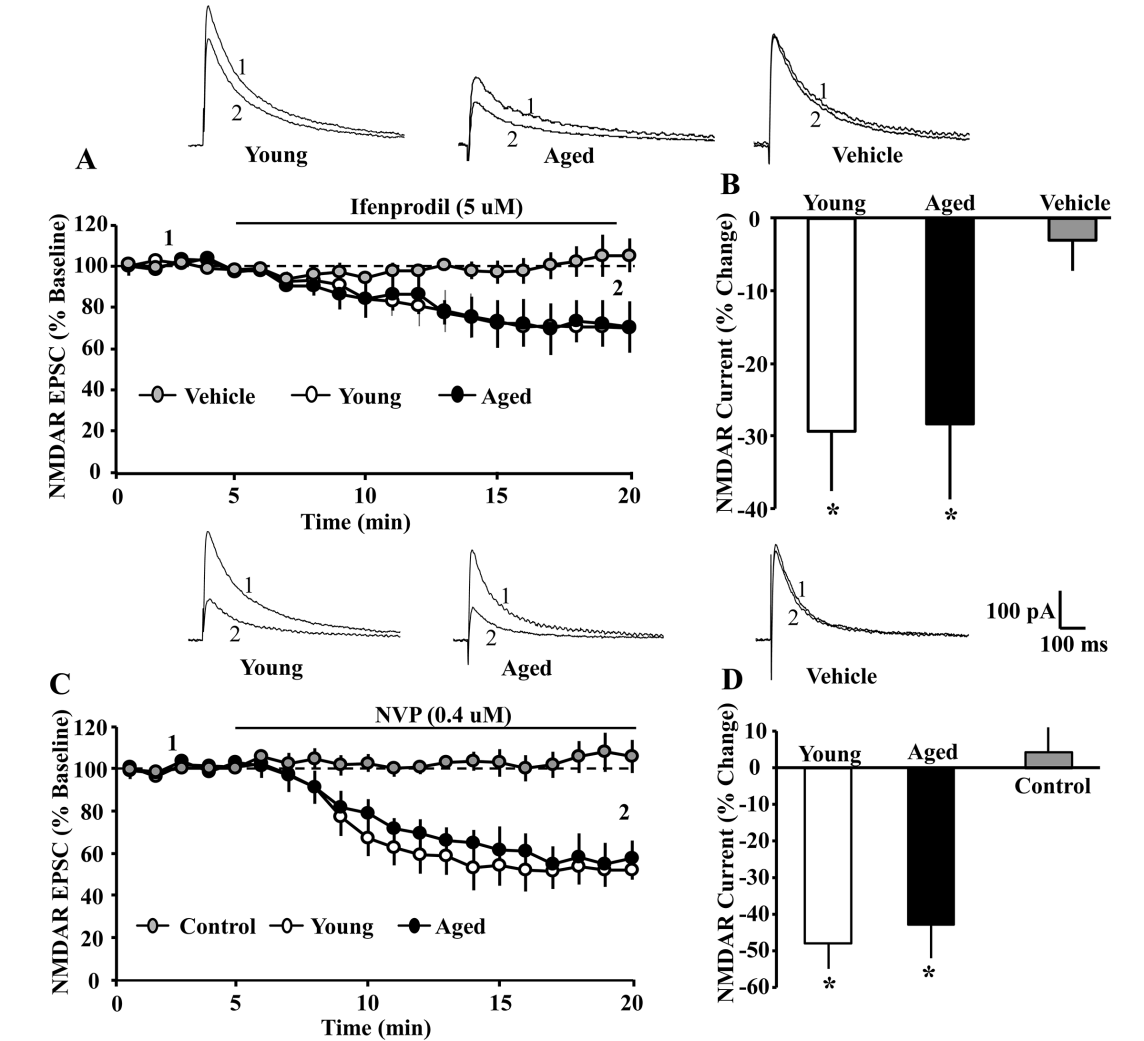
**The GluN2A and GluN2B selective antagonists attenuated the NMDAR EPSC amplitude to a similar extent in young and aged CA1 pyramidal neurons.** For each cell, the peak response was normalized to the 5 min pre-drug baseline. (**A**) Time course of the decrease in the NMDAR EPSCs recorded from CA1 hippocampal pyramidal neurons 5 min before and 15 min after bath application of ifenprodil (5 µM, solid line) in young (open circle, n = 4/4 cells/animals) and aged (filled circle, n = 6/5 cells/animals) animals. For the control condition (gray circle, n = 7/6 cells/animals, young-aged combined) recordings were obtained before and after application of ethanol vehicle. (**B**) Bar graph demonstrates percentage decrease in NMDAR EPSCs for young and aged animals following application of ifenprodil or vehicle. Asterisks indicate a significant difference from baseline. The top panel provides representative traces illustrating the NMDAR EPSC at baseline (1) and at the end of a 15 min of ifenprodil application (2) recorded from a young (left) or aged (middle) cell, and for a cell recorded in the vehicle control condition (right). The GluN2A selective antagonist, NVP, attenuated the NMDAR EPSC to a similar extent in young and aged CA1 pyramidal neurons. For each cell, the peak response was normalized to the 5 min pre-drug baseline. (**C**) Time course of the decrease in the NMDAR EPSCs recorded from CA1 hippocampal pyramidal neurons 5 min before and 15 min after bath application of NVP (0.4 µM, solid line) in young (open circle, n = 4/4 cells/animals) and aged (filled circle, n = 5/5 cells/animals) animals. For the control condition (gray circle, n = 6/6 cells/animals, young-aged combined) recordings were maintained for the same duration in the absence of NVP application. (**D**) Bar graph demonstrates percentage decrease in NMDA EPSCs during the last 5 min of recording. Asterisks indicate a significant difference from baseline. Representative traces on the top illustrating the NMDAR EPSC at baseline (1) and at the end of a 15 min NVP application (2) recorded from a young (left) or aged (middle) cell, and for a cell in the control condition (right).

Next, we employed GluN2 selective antagonists to examine age differences in the contribution of GluN2 subtypes to the EPSC peak synaptic response. Again, cells were held at +40 mV. Following at least five-minutes of stable baseline recording of the isolated NMDAR EPSC, the GluN2B selective antagonist, ifenprodil (5 µM), was bath applied. Ifenprodil decreased the peak EPSC by ~30%, and an ANOVA on percent of baseline for the peak EPSC, measured 15 min following application of ifenprodil, indicated no age difference (young: 70.59 ± 8.14 mean ± SEM% of baseline, n = 4/4 cells/animals; aged: 71.58 ± 10.39%, n = 6/5 cells/animals) (Fig. 2 A & B). The decrease was specific to ifenprodil, as no effect was observed following application of vehicle alone.

Similarly, bath application of the GluN2A selective antagonist, NVP (0.4 µM), reduced the peak NMDAR EPSC by ~45% over the next 15 min. No change in the peak EPSC was observed in the absence of NVP, recorded over the same duration. An ANOVA on percent of baseline for the peak EPSC, measured 15 min following application of NVP, indicated no age difference in the reduction of the EPSC (young: 52.10 ± 7.28% of baseline, n = 4/2 cells/animals; aged: 57.14 ± 9.2%, n = 6/3 cells/animals) (Fig. 2 C & D).

### The DTT-mediated potentiation in NMDAR currents involves an increased contribution of GluN2B

**Figure 3:**
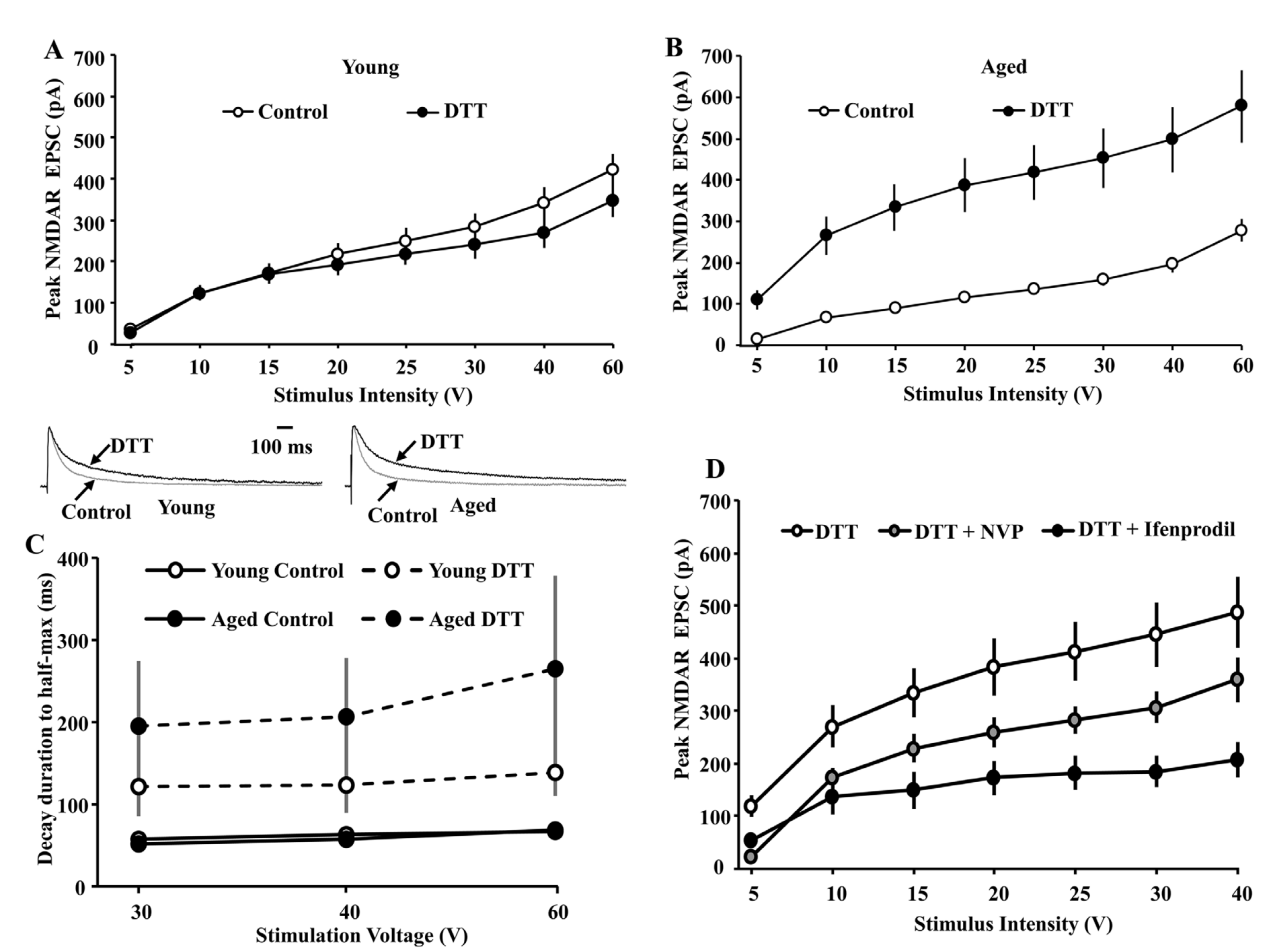
**Input-output curves examining age-related differences in the peak NMDAR EPSCs under control conditions and in the presence of DTT (0.5 mM).** The cells were voltage clamped at +40 mV. (**A**) Bath application of DTT (filled circle, n = 8/2 cells/animals) failed to increase NMDAR EPSCs for CA1 pyramidal cells recorded from young animals relative to the control condition (open circle, n = 25/11 cells/animals). (**B**) Across the range of stimulation intensities, DTT (filled circle, n = 7/3 cells/animals) significantly augmented NMDAR EPSCs in CA1 cells recorded from aged animals relative to the control condition (open circle, n = 26/14 cells/animals). (**C**) DTT increases the time to half-decay of the NMDAR synaptic response. The symbols represent the mean (±SEM) time of NMDAR-mediated EPSC to decay to 50% of the peak under control conditions and in the presence of DTT for the three highest stimulation intensities. The inset (left) shows time course of the EPSC, evoked by 40 V stimulation, across all CA1 pyramidal cells recorded from young animals under the control condition (gray trace, n = 20/9 cells/animals) and in the presence of DTT (black trace, n = 8/2 cells/animals). The inset (right) time course of the EPSC, evoked by 40 V stimulation, across all CA1 pyramidal cells recorded from aged animals under the control condition (gray trace, n = 26/14 cells/animals) and in the presence of DTT (black trace, n = 7/3 cells/animals). For each cell, the response amplitude evoked by 40 V stimulation was normalized to the peak of the response. (**D**) Increased contribution of the GluN2B subunit to the peak NMDAR EPSC following DTT-induced potentiation of NMDAR EPSCs in slices obtained from aged animals. The cells were voltage clamped at +40 mV and input-output curves of the peak NMDAR EPSCs were generated in presence of DTT (open circle, n = 7/3 cells/animals), DTT+NVP (gray circle, n = 5/2 cells/animals), and DTT+ifenprodil (filled circle, n = 5/3 cells/animals).

Following bath application of the reducing agent DTT (0.5 mM), input-output curves of the peak NMDAR EPSC were again obtained (aged: n = 7/3 cells/animals, young: n = 8/2 cells/animals) and compared to input-output curves for the respective control condition for young and aged animals (Fig. 1C). A repeated measures ANOVA across stimulation intensities for the control condition and DTT conditions, within each age group, indicated an effect of treatment on the peak EPSC for aged animals [F(1,203) = 53.24, p < 0.0001], but not for young animals [F(1,217) = 0.49, p > 0.05] (Fig. 3A & B), confirming an age-related difference in the DTT-induced potentiation of the NMDAR response.

To determine if DTT influenced the decay rate of the NMDAR response, the NMDAR EPSC in response to 30, 40, and 60 V stimulation was normalized to the peak of the response at each stimulation intensity, and the time to decay to 50% of the peak was calculated in the presence of DTT and compared to the control condition. Bath application of DTT increased the time to half-decay ~4 fold in aged animals and ~2 fold in young animals (Fig. 3 C). An ANOVA repeated across stimulation intensities indicated a treatment effect [F(1,106) = 22.76, p < 0.0001] in the absence of an age difference or an interaction of age and treatment. ANOVAs within each age group confirmed that DTT increased the time to half-decay of the EPSC for aged [F(1,52) = 12.61, p < 0.005] and young [F(1,54) = 11.55, p < 0.005] animals.

To examine the contribution of GluN2A/GluN2B subunit to DTT-induced potentiation in NMDAR function, subunit selective antagonists were applied to slices obtained from aged animals in the presence of DTT. Figure 3D shows input-output curves collected from cells of aged animals exposed to DTT alone (n = 7/3 cells/animals), or in the presence of DTT followed by application of NVP (n = 5/2 cells/animals) or ifenprodil (n = 5/3 cells/animals). A repeated measures ANOVA across stimulation intensities indicated an interaction of stimulation intensities and treatment [F(12,84) = 5.01, p < 0.0001]. Post hoc comparisons for treatment effects indicated NVP tended (p = 0.055) to decrease the peak response to ~65%, relative to the DTT alone condition. The effect of ifenprodil was more robust, decreasing the peak response (p < 0.005) to ~45% of the DTT alone condition (Fig. 3D). Accordingly, in contrast to the greater effect of NVP under controls conditions, ifenprodil reduced the peak response to a greater extent in the presence of DTT. Thus, DTT application increased decay time and increased sensitivity to ifenprodil in aged animals, suggest an increase contribution of GluN2B to the NMDAR synaptic response.

### Redox regulation of NMDAR function depends on NMDAR activity

**Figure 4:**
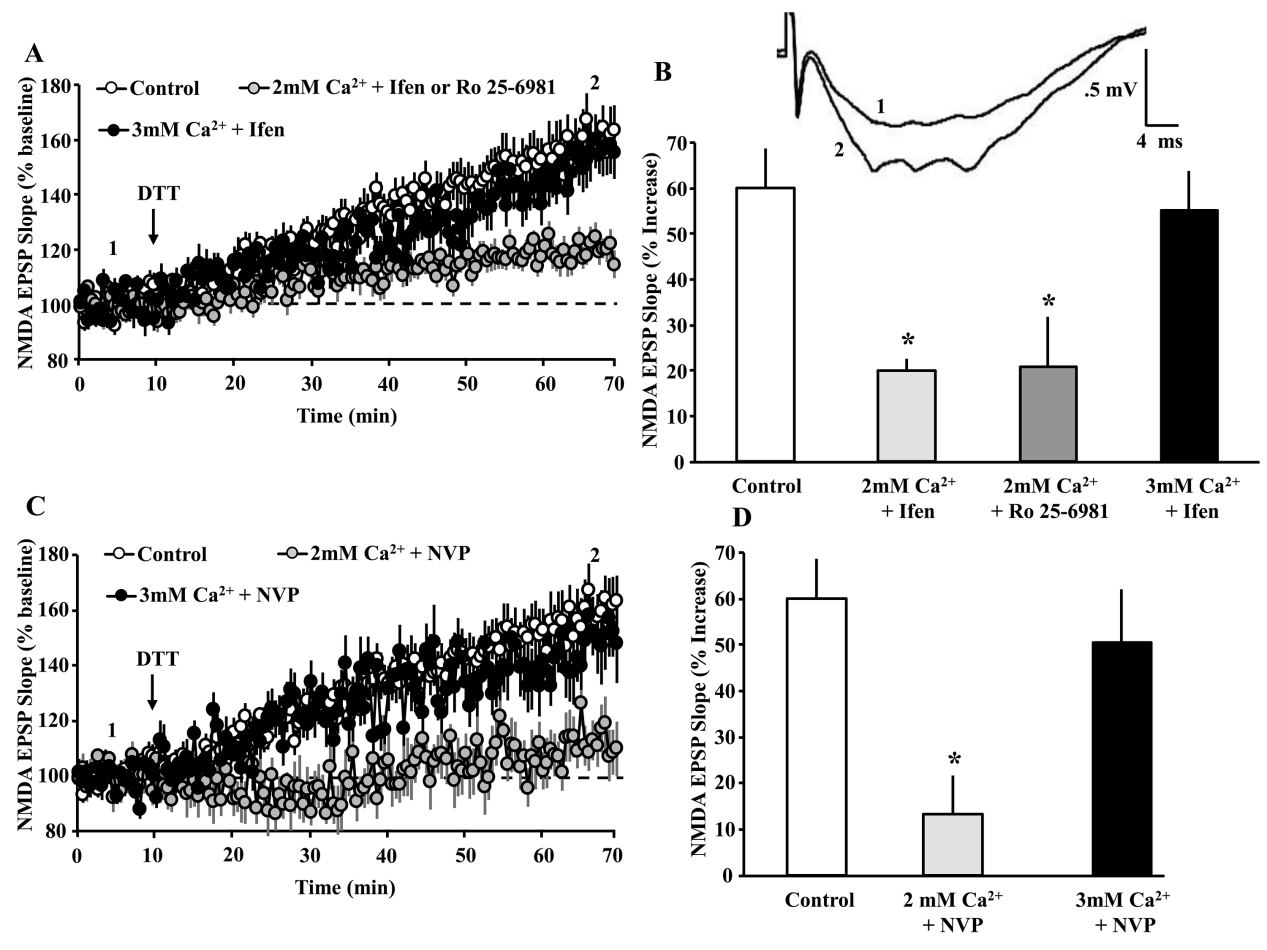
**NMDAR activity and Ca^2+^ are required for the DTT-induced potentiation of NMDAR synaptic function.** (**A**) Time course of mean (±SEM) NMDAR-fEPSP slope normalized to the baseline (dashed line) for the control condition (open circles), in the presence of ifenprodil or Ro 25-6981 in 2 mM Ca^2+^ recording medium (gray circles), and ifenprodil in 3 mM Ca^2+^ recording medium (filled circles). For clarity, the responses for the GluN2B antagonists (ifenprodil and Ro 25-6981) in 2 mM Ca^2+^ recording medium were combined. The arrow indicates the time of DTT (0.5 mM) application. The insert provides an example of the growth of the NMDAR-mediated fEPSP during baseline (1) and 60 min following application of the DTT (2) under the control condition. (**B**) Bar graph demonstrates the percent change in NMDAR-mediated fEPSP response during the last 5 min of recording, due to DTT application under the control condition (open bar, n = 31/26 slices/ animals), 2 mM Ca^2+^ + ifenprodil (light gray bar, n = 8/8 slices/animals), 2 mM Ca^2+^ + Ro 25-6981 (gray bar, n = 5/5 slices/animals), and 3 mM Ca^2+^ + ifenprodil (black bar, n = 8/4 slices/animals). (**C**) Time course of mean (±SEM) NMDAR-fEPSP slope normalized to the baseline (dashed line) for the control condition (open circles), in the presence of NVP in 2 mM Ca^2+^ recording medium (gray circles), and NVP in 3 mM Ca^2+^ recording medium (filled circles). The arrow indicates the time of DTT (0.5 mM) application. (**D**) Bar graph demonstrates the percent change in NMDAR-mediated fEPSP response during the last 5 min of recording, due to DTT application under the various conditions including control (open bar, n = 31/26 slices/animals), NVP (light gray bar, n = 7/6 slices/animals), and 3 mM Ca^2+^ + NVP (black bar, n= 8/4 slices/animals). For B & D, the asterisks indicate a significant difference relative to control.

Next, we sought to determine if the DTT-mediated growth of the NMDAR response in aged animals represents an activity-dependent NMDAR plasticity. An influx of Ca^2+^ following NMDAR activation induces long-term modifications in NMDAR function in young animals [34,35]. Due to the inhibition of Ca^2+^ entry for cells held at +40 mV, we employed extracellular fEPSP recordings of isolated NMDAR synaptic responses to examine the role of NMDAR activity and Ca^2+^ on the DTT-mediated potentiation of the NMDAR synaptic response in slices from aged animals. Control recordings, examining the effects of DTT alone, were interleaved between recordings in which the GluN2A or GluN2B selective antagonist were applied, prior to DTT application. For control slices (n = 31slices/26 aged animals), DTT (0.5 mM) induced an increase (161.2 ± 6.2% mean ± SEM of baseline) in the NMDAR-mediated synaptic response, similar to previous reports [6,8,9,13] (Fig. 4).

The GluN2B antagonists, ifenprodil (5 µM, n = 8/8 slices/animals) or Ro 25-6981 (5 µM, n = 5/5 slices/animals) were added to the bath at least 45 min prior to application of DTT. In another set of slices (8 slices from 4 animals), the concentration of Ca^2+^ in the bath was increased from 2 mM to 3 mM, and ifenprodil was added to the bath at least 45 min prior to application of DTT. An ANOVA on the percent increase in the synaptic response, 60 min following application of DTT indicated a treatment effect [F(3,48) = 5.7, p = 0.02]. Post hoc tests indicated that, in 2 mM Ca^2+^ recording medium, ifenprodil (120.0 ± 2.5%) and Ro 25-6981 (120.8 ± 10.0%) inhibited the DTT-induced growth of the NMDAR-mediated synaptic responses, relative to the control condition (Fig. 4 A & B). In contrast, when the level of Ca^2+^ was increased to 3 mM, ifenprodil failed to attenuate the DTT-induced potentiation of NMDAR-mediated synaptic responses (155.2 ± 8.7%) (Fig. 4 A & B).

To determine the contribution of GluN2A to the DTT-induced potentiation of the NMDAR response, NVP (0.4 µM, n = 7/6 slices/animals) was added to the bath 45 min prior to application of DTT. For some slices (n = 8/4 slices/animals), the concentration of Ca^2+^ in the bath was increased from 2 mM to 3 mM, and NVP was added to the bath 45 min prior to application of DTT. An ANOVA on the percent change in the response, 60 min after DTT application, indicated a difference across treatment groups [F(3,45) = 4.48, p = 0.0078]. Post hoc tests indicated that NVP in 2 mM Ca^2+^ significantly attenuated the DTT-mediated growth (113.3 ± 6.4%) relative to controls (Fig. 4 C & D). Again, raising the level of Ca^2+^ was able to overcome the NMDAR antagonist inhibition of the DTT-mediated growth in the synaptic response. Together, the results indicate that the DTT-mediated growth of the NMDAR response depends on the level of NMDAR activation and Ca^2+^ entry.

**Figure 5:**
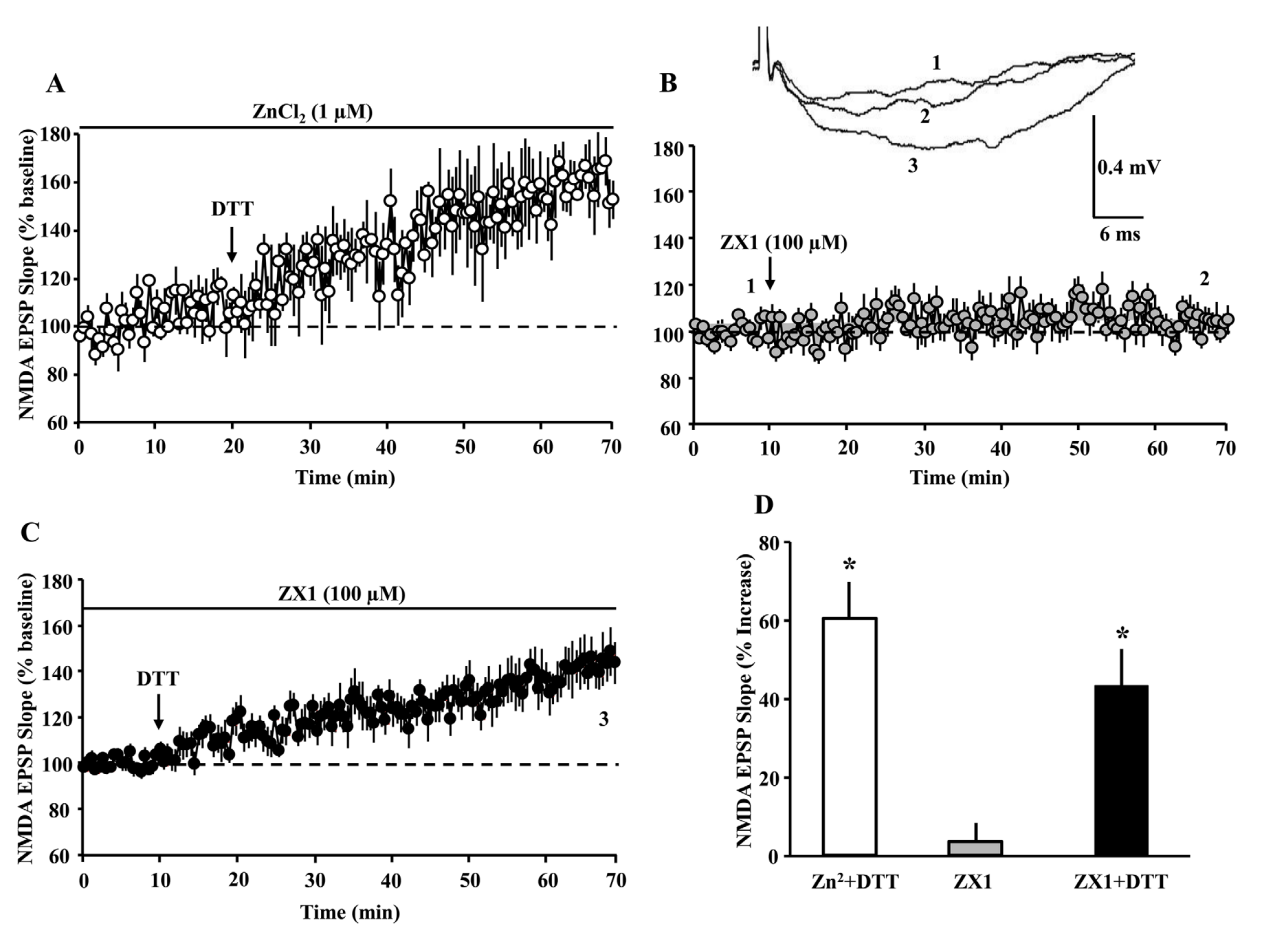
**The DTT-induced potentiation of the NMDAR-synaptic response is not due to zinc chelation.** The panels A-C illustrate the time course for the NMDAR-fEPSP slope; each point represents the mean (±SEM), normalized to the baseline (dashed line). (**A**) The arrow indicates the time of bath application of DTT (0.5 mM) in presence of ZnCl_2_ (1 µM). (**B**) The arrow indicates the time of bath application of ZX1 (100 µM). (**C**) The last ten min of NMDAR-fEPSP slope recording in presence of ZX1 was renormalized and DTT was added (arrow). (**D**) Bar graph represents the mean (+SEM) percent change in NMDAR-mediated fEPSP during the last 5 min of recording, in response to Zn^2+^ plus DTT (open bar, n = 3/3 slices/animals), ZX1 alone (gray bar, n = 11/6 slices/animals,) and ZX1 plus DTT (filled bar, n = 11/6 slices/animals). Asterisks indicate significant potentiation relative to baseline.

One possibility is that DTT increased Ca^2+^ entry through GluN2A containing receptors by chelating Zn^2+^ [24,36–38]. However, addition of excess Zn^2+^ (ZnCl_2_ 1 µM, n = 3/3 slices/animals) failed to block the effects of DTT, which increased the response (160.3 ± 8.4%) (Fig. 5A). In addition, bath application of the Zn^2+^ chelating agent ZX1 (100 µM, n = 11 slices/6 animals) failed to enhance the NMDAR synaptic response (103.5 ± 3.99%) (Fig. 5B). In contrast, when DTT was subsequently applied 60 min following application of ZX1, a significant (p < 0.0001) increase (143.6 ± 8.79%, n = 11 slices/6 animals) in NMDAR response was observed, suggesting that DTT effects are not due to Zn^2+^ chelation (Fig. 5C).

**Figure 6:**
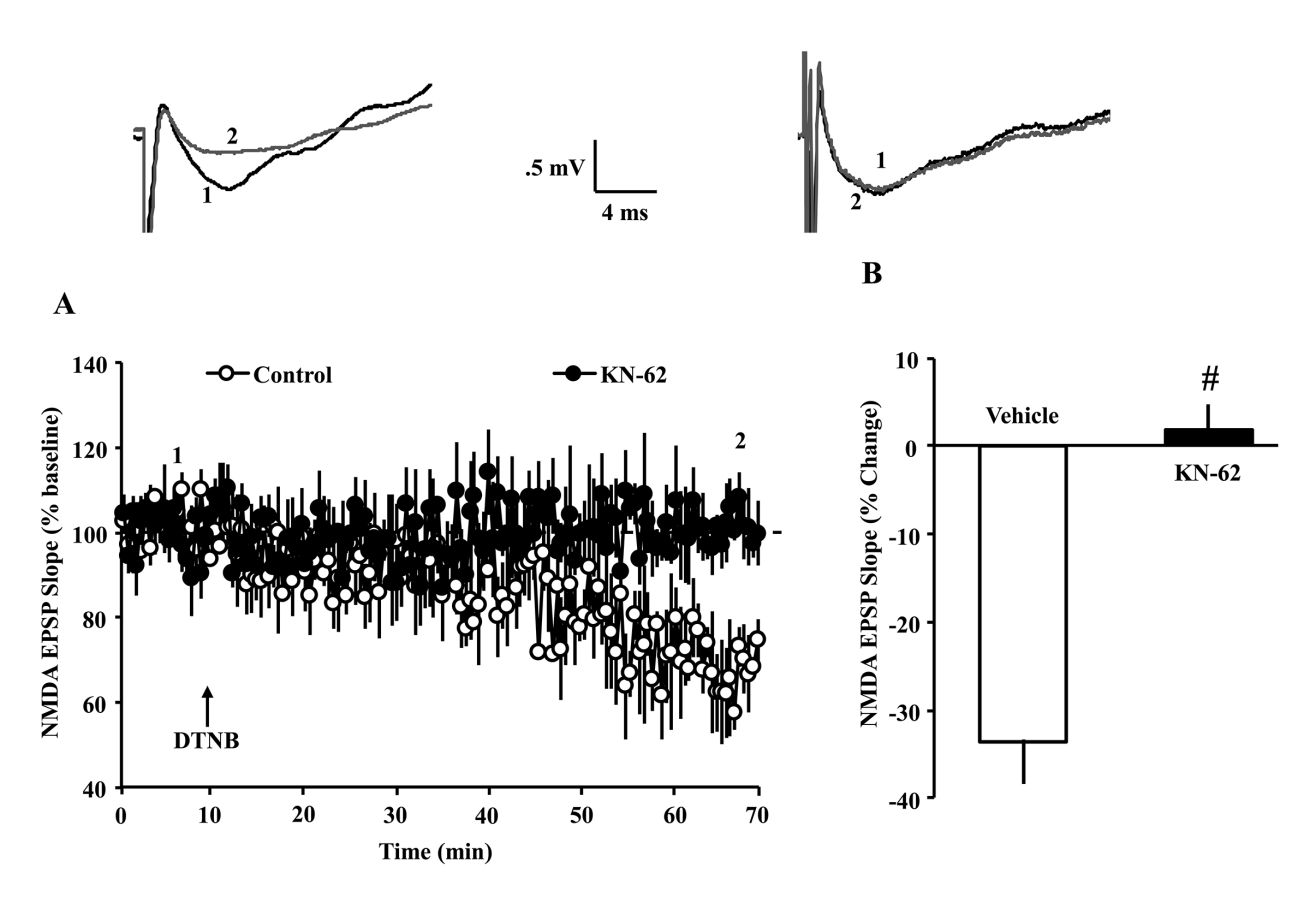
**Decrease in NMDAR synaptic responses in young animals, under oxidizing conditions, depends on CaMKII activity**. (**A**) Time course of normalized NMDAR-fEPSP slope following application of DTNB (0.5 mM, arrow) in the young animals. Each point represents the mean (±SEM), normalized to the baseline (dashed line), for slices in the control condition (open circles) or following pre-incubation with the CaMKII inhibitor, KN-62 (10 µM, filled circles). DTNB reduced NMDAR synaptic response. Pre-incubation with KN-62 blocked the decrease in the NMDAR response associated with DTNB application. (**B**) Quantification of the mean percent change in the NMDAR-fEPSP slope during the last 5 min of recording in the presence of vehicle control (open, n = 7/7 slices/animals) and KN-62 (, n = 7/7 slices/animals). Pound sign indicates a significant difference between the two groups. The waveforms represent examples of NMDAR-fEPSPs recorded during baseline (1) and 60 min following application of DTNB (2) in the control condition (left) and following pre-incubation in KN-62 (right).

In young animals, influx of Ca^+2^ through NMDARs results in CaMKII-mediated trafficking and insertion of NMDAR subunits into the postsynaptic membrane [27,28], and the DTT-mediated increase in the NMDAR response in older animals is blocked by inhibition of CaMKII [6]. In addition, CaMKII activity determines the long-term maintenance of synaptic strength [32,39]. Oxidizing agents decrease the NMDAR synaptic response, specifically in young animals [6,14,40]. To determine whether ongoing CaMKII activity is involved in the decrease of NMDAR responses under oxidizing conditions, slices from young animals were exposed to the CaMKII inhibitor KN-62 (10 µM) or dimethyl sulfoxide (DMSO) vehicle for at least 60 min before application of the oxidizing agent, 5,5′-dithiobis(2-nitrobenzoic acid) (DTNB) (0.5 mM). Bath application of DTNB in the presence of vehicle decreased the NMDAR-fEPSP (66.3 ± 4.9%, 7/7 slices/animals) 60 min following drug application. The DTNB-induced decrease in the NMDAR-fEPSP was blocked by the CaMKII selective antagonist, KN-62 (101.6 ± 2.9%, 7/7 slices/animals) [F(1,12) = 37.97, p < 0.0001], suggesting that the basal level of kinase activity determines the effectiveness of redox modulators on the NMDAR response (Fig. 6 A & B).

## DISCUSSION

An understanding of the mechanisms for regulation of NMDAR function is important, due to the critical role of NMDARs in synaptic plasticity and cognition. Redox-mediated NMDAR hypofunction is linked to cognitive deficits for a range of illnesses including Alzheimer’s disease, depression, and schizophrenia [4,41,42]. However, the molecular mechanism for redox regulation of NMDARs in aging and disease is unknown. The age-related decrease in the NMDAR synaptic response is well characterized and contributes to altered synaptic plasticity and impaired cognition [15–18]. Recent studies point to redox regulation as a mechanism contributing to the decline in NMDAR function with age [6,8,11,12,43].

On possible mechanism involves a shift in the subunit composition during aging, which would determine the ability of redox reagents to modify NMDAR function [24,44]. NMDARs are heterotetramers and much of the previous research has focused on diheteromeric NMDARs with two GluN1 subunits and two identical GluN2 subunits, either GluN2A or GluN2B. The diheteromeric GluN2 subunits have different kinetics and are differentially sensitive to Zn^2+^ and redox reagents. The GluN2A subunit is more sensitive to Zn^2+^ inhibition and has unique extracellular cysteine residues. Under oxidizing conditions, S-nitrosylation of cysteine residues or disulfide bonds between pairs of cysteine residues decreases receptor function [21,23,24]. During the first two weeks of postnatal development, the synaptic GluN2B subunits may be switched for GluN2A subunits [45]. If this switch continues with advancing age, metabolic oxidative stress during aging could act on extracellular cysteine residues of GluN2A to decrease NMDAR function and increase responsiveness to reducing agents. In contrast, the results of the current study suggest that the ratio of diheteromeric GluN2A/GluN2B receptors remain stable with advancing age. An increase in the proportion of GluN2A subunits would decrease the decay time, shortening the synaptic response [25,33]. Conversely, we found that the decay of the synaptic response was not different between young and aged animals. In addition, the proportional decrease in the NMDAR EPSCs, in response to selective GluN2 antagonists, was similar for young and aged animals, indicating that diheteromeric GluN1-GluN2A receptors did not increase with age.

Previous work suggests that the majority of NMDARs in the hippocampus are triheteromeric, having two different GluN2 subunits (GluN1/GluN2A/GluN2B) [37,46,47]. Indeed, under control conditions, ifenprodil and NVP decreased the synaptic response by ~30% and ~45%, respectively, which is similar to previous reports examining cells that express a combination of diheteromeric and triheteromeric NMDARs [37,47–49]. The absence of an age difference for the synaptic decay and the similarity in the response inhibition by GluN2 selective antagonists suggest that the proportion of GluN2A/GluN2B subunits is not altered with advancing age. This conclusion is consistent with studies that examine expression of both GluN2A and GluN2B subunits in the same animal. When both subunits are examined, GluN2A and GluN2B are observed to decline equally during aging [50,51]. Together, the results suggest that the age difference in NMDAR synaptic function and redox sensitivity does not involve a large shift in the expression ratio of GluN2A/GluN2B at the synapse. Nevertheless, it is still possible that the decrease in the NMDAR responses and increased sensitivity to DTT involves redox regulation of extracellular cysteine residues on GluN1 or GluN2A [21]. However, extracellular application of glutathione, which can potentiate GluN1-GluN2A channels, does not increase the NMDAR response in older animals [6,21]. Finally, if the age difference was due to redox of GluN2A subunits, then we would expect that DTT would specifically increase the contribution of GluN2A subunits to the synaptic response. In contrast, the results of the current study indicate that GluN2B is a major contributor to the DTT-induced potentiation of the NMDAR response.

DTT markedly increased the decay time of NMDAR EPSCs in cells from young and aged animals. The increased in the decay time is consistent with an increased contribution of GluN2B [25,33,46,52,53]. An increased contribution of GluN2B was also evident in the effectiveness of selective GluN2 antagonists in reducing the peak response. The ifenprodil-induced decrease in the peak response of aged animals was greater in the presence of DTT, reducing the EPSC to ~45% of baseline, relative to the control condition, which was ~70% of baseline. Similarly, the contribution of GluN2A to the synaptic currents diminished following application of DTT. NVP deceased the EPSC peak response to ~55% of baseline in the control condition and to ~65% of baseline in the presence of DTT. The results are consistent with a DTT-mediated increase in the contribution of GluN2B, particularly for aged animals.

In younger animals, an NMDAR activity-dependent long-term potentiation (LTP) of NMDAR synaptic transmission involves trafficking of NMDARs to the synapse [54]. The mechanism for expression of LTP of NMDAR function and redox regulation of NMDAR function across the lifespan may involve similar mechanisms. In both cases, the increase in the synaptic response is not associated with a change in paired-pulse facilitation, and does not require an increase in the α-amino-3-hydroxy-5-mehtyl-4-isoxazolepropionic (AMPA) receptor component of synaptic transmission [6,34,35]. The results suggest that expression of LTP of NMDAR and DTT-mediated potentiation are not due to presynaptic changes.

Postsynaptic Ca^+2^ influx through NMDARs, results in CaMKII-mediated trafficking and insertion of NMDAR subunits into the postsynaptic membrane [27,28], modifying the NMDAR subunit composition at the synapse [55,56]. Furthermore, activated CaMKII associates with GluN2B subunit, increasing the contribution of GluN2B to NMDAR function [28–32]. Similar to activity-induced potentiation of NMDAR synaptic transmission, the DTT-induced growth of the NMDAR synaptic response is also CaMKII-dependent [6] and our current results point to an increased contribution of GluN2B to the NMDAR response.

The induction of LTP of NMDARs requires NMDAR activity and Ca^2+^ influx [34,35,57–61]. Using selective antagonists and different levels of extracellular Ca^2+^, we observed that the DTT-mediated increase in the NMDAR response was also dependent on NMDAR activity and level of Ca^2+^. The results suggest that the DTT-mediated increase in NMDAR function in aged animals involves a redox-mediated increase in the activity of CaMKII to activate mechanisms similar to LTP of NMDARs. As such, redox regulation of CaMKII activity may contribute to inhibition of LTP of NMDAR function under oxidizing conditions [14] and impaired LTP of NMDAR function and NMDAR trafficking in advanced age [62].

In the current study, we observed that prior inhibition of CaMKII in young animals blocked a decrease in the NMDAR synaptic response under oxidizing conditions, suggesting that the redox-CaMKII pathway is also important for the redox-mediated decrease in NMDAR function. As such, the redox-CaMKII pathway may contribute to the age-related difference in basal NMDAR function in a manner similar to CaMKII regulation of the maintenance of synaptic strength [32,39]. However, more research is required to determine if the decrease response, due to oxidizing agents, involves a differential influence on GluN2A and GluN2B. CaMKII activity is involved in regulated trafficking and synaptic insertion of GluN2A subunits [27,28,63].

Redox mediated NMDAR hypofunction can act as metaplasticity mechanism, regulating synaptic modifiability required for synaptic networks that underlie cognition [2,3,64]. Furthermore, redox-mediated NMDAR hypofunction, and the interaction of CaMKII and GluN2B are thought to provide a link between altered synaptic plasticity and cognitive deficits for a range of illnesses including Alzheimer’s disease, depression, schizophrenia, and impaired episodic memory during aging [4,8,10,41,42,65,66]. Thus, therapeutic interventions to alleviate the redox-mediated NMDAR hypofunction may rescue synaptic plasticity and improve cognitive function during aging and neurological or psychiatric diseases.

## MATERIALS AND METHODS

### Animals

Procedures involving animals were reviewed and approved by the Institutional Animal Care and Use Committee of University of Florida and were in accordance with guidelines established by the U.S. Public Health Service Policy on Humane Care and Use of Laboratory Animals. Male Fischer 344 rats, young (4–6 months, n = 30) and aged (24-26 months, n = 95), were obtained from the National Institute on Aging colony at Harlan (Indianapolis, IN, USA).

### Whole Cell Patch Clamp Recordings

Rats were anaesthetized with intraperitoneal injection of xylazine (10 mg/kg) and ketamine (100 mg/kg). Isoflurane (5%) was administered for 5 min, and following no indication of a withdrawal reflex, animals were perfused transcardially with an ice-cold sucrose-cutting solution (~50 ml) containing (in mM): 206 sucrose, 2 KCl, 25 NaHCO_3_, 1.2 NaH_2_PO_4_, 1 CaCl_2_, 1 MgSO_4_, 0.01 glycine, and 10 D-glucose saturated with 95% O_2_/5% CO_2_. Following decapitation, brains were removed and placed in ice-cold sucrose-cutting solution for 2 minutes. Horizontal hippocampal sections of 350 µm were generated using a Lecia vibratome 3000 (Buffalo Grove, IL, USA) and sections were immediately transferred to a holding chamber with artificial cerebrospinal fluid, at 32-36ºC, containing (in mM): 124 NaCl, 2.5 KCl, 25 NaHCO_3_, 1.23 NaH_2_PO_4_, 1 CaCl, 3 MgSO_4_, and 10 D-glucose saturated with 95% O_2_/5% CO_2_. Sections were allowed to equilibrate for at least 30 min prior to being transferred into the recording chamber. The recording chamber was perfused (2ml/min) with recording medium (in mM): 126 NaCl, 3 KCl, 25 NaHCO_3_, 1.2 NaH_2_PO_4_, 2.4 CaCl_2_, 1.5 MgSO_4_, and 11 D-glucose, saturated with 95% O_2_/5% CO_2_, warmed to 30ºC. Prior to patch-clamp recording, sections were allowed to equilibrate for at least 20-minute in the recording chamber.

The recording chamber was mounted on a Burleigh-Gibarltar stage (Thor labs, Newton, NJ, USA) attached to an Olympus BX51WI microscope with infrared differential interference contrast optics (Shinjuku, Tokyo, Japan). Sections were visualized at 10x using a Hamamatsu C4742-95 digital camera (Hamamatsu City, Shizuoka Pref., Japan) attached to a Dell Optiplex 7010 running HCImage Live software (Hamamatsu) on a Windows 7 OS. Glass micropipettes (Sutter, Novato, CA, USA) were filled with a CsMeS0_3_- based solution containing (in mM): 140 CsMeS0_3_, 8 NaCl, 1 MgCl_2_, 0.2 EGTA, 10 HEPES, 2 Mg-ATP, 0.3 Na-GTP, 5 QX-314, pH 7.3 (CsOH). NMDAR EPSCs were isolated by addition of 6,7-dinitroquinoxaline-2,3-dione (DNQX, 30 μM, Cayman Chemical) and picrotoxin (PTX, 20 μM, Tocris) to the recording medium. Open-tip glass pipette resistances ranged from 3-6 MΩ. Pipettes were placed directly over CA1 pyramidal cell bodies at 10x (~0.2mm from the stimulator electrode) and then a 40x water-emersion objective was used to visualize contact with cell membranes. Whole-cell voltage clamp recordings were obtained with an Axon Multiclamp700B (Molecular Devices, Sunnyvale, CA, USA). Signals were sampled at 10kHz, filtered at 1kHz, and digitized with a Digidata 1440A (Molecular Devices) using Clampex (v.10.2, Molecular Devices). Cells were held at -60mV while access resistance, membrane resistance, and whole-cell capacitance were measured using a -10mV step protocol. Average whole-cell parameters are presented in Table 1. Cells were not included in the final analyses if access resistance changed more than ~35% during the experiment. A concentric bipolar stainless steel electrode was positioned in stratum radiatum and 50 µsec bipolar pulses (0.033 Hz) from a Grass stimulator (SD9) were used to evoke EPSCs and generate stimulation voltage-peak EPSC curves. For pharmacological studies, stimulation intensity was adjusted in order to obtain ~50% maximum EPSC and a baseline response was recorded prior to drug application.

### Extracellular field potential recordings

Methods for hippocampal slice preparation and electrophysiological recording of total and NMDAR-mediated synaptic responses have been published previously [6,8]. Briefly, hippocampi were harvested and slices (~400 µm) cut parallel to the alvear fibers. Slices were placed in a recording chamber and bathed in 30 ± 0.5^o^C oxygenated recording medium (in mM): NaCl 124, KCl 2, KH_2_PO_4_ 1.25, MgSO_4_ 2, CaCl_2_ 2, NaHCO_3_ 26, and glucose 10. Extracellular fEPSP from stratum radiatum of CA1 were recorded with glass micropipettes (4-6 MΩ) filled with recording medium. A concentric bipolar stainless steel electrode was positioned ~1 mm away in the middle of the stratum radiatum. Field potentials (0.033 Hz) were evoked by biphasic stimulus pulses (100 μsec). Signals were amplified, filtered (1 Hz and 1 kHz), and stored on computer for off-line analysis. For analysis, two cursors were placed around the initial descending phase of the waveform and the maximum slope (mV/ms) of the fEPSP was determined by a computer algorithm that found the maximum change across all sets of consecutively recorded points (20 kHz sampling rate) between the 2 cursors.

The NMDAR-mediated component of synaptic transmission (NMDAR-fEPSP) was isolated by incubating slices in recording medium containing low Mg^2+^ (0.5 mM), DNQX (30 μM), and PTX (10 μM) for at least 60 minutes. For pharmacological studies, the stimulation intensity was adjusted to evoke a response ~50% of maximum and baseline NMDAR-mediated synaptic responses were recorded for at least 10 min prior to and for 60 min after drug application.

### Pharmacological agents

Other pharmacological agents were bath applied at final concentrations of: DTT (0.5 mM, Sigma), DTNB (0.5 mM, Sigma), 2-amino-5-phosphonopentanoic acid (AP5, Sigma, 100 µM), NVP-AAM077 (NVP, 0.4 µM, Sigma), ifenprodil (5 µM, Sigma), Ro 25-6981 (5 µM, Tocris), ZnCl_2_ (1 µM, Sigma), ZX1 (100 µM, Strem Chemicals), and 4-[(2*S*)-2-[(5-isoquinolinylsulfonyl)-methylamino]-3-oxo-3-(4-phenyl-1-piperazinyl)propyl] phenyl isoquinolinesulfonic acid ester (KN-62, 10 µM, Tocris). DTT, Ro 25-6981, NVP, ZnCl_2_, and ZX1 were directly dissolved in the recording medium. DNQX was initially dissolved in DMSO (Sigma) and diluted in recording medium to a final DMSO concentration of <0.01%. PTX, DTNB, and ifenprodil were dissolved in ethanol and diluted in recording medium to a final ethanol concentration of 0.0001%.

### Statistical analysis

ANOVAs were carried out using StatView 5.0 (SAS Institute) in order to determine significant main effects and interactions. Post hoc ANOVAs and Fisher’s protected least significant difference comparisons, with the p-value set at 0.05, were used to further localize significant differences. In cases of multiple comparisons (e.g. baseline responses relative to DTT or NMDAR antagonists), Bonferroni corrections were applied.

## SUPPLEMENTARY MATERIAL

Supplementary Figures
