# Supplementary material for: Subunit contribution to NMDA receptor hypofunction and redox sensitivity of hippocampal synaptic transmission during aging
Source: Aging (Albany NY). 2019 Jul 24;11(14):5140–57. doi: 10.18632/aging.102108 (PMC6682512; doi:10.18632/aging.102108)
Supplement: Supplementary Figures [file aging-11-102108-s001.pdf]

SUPPLEMENTARY MATERIAL

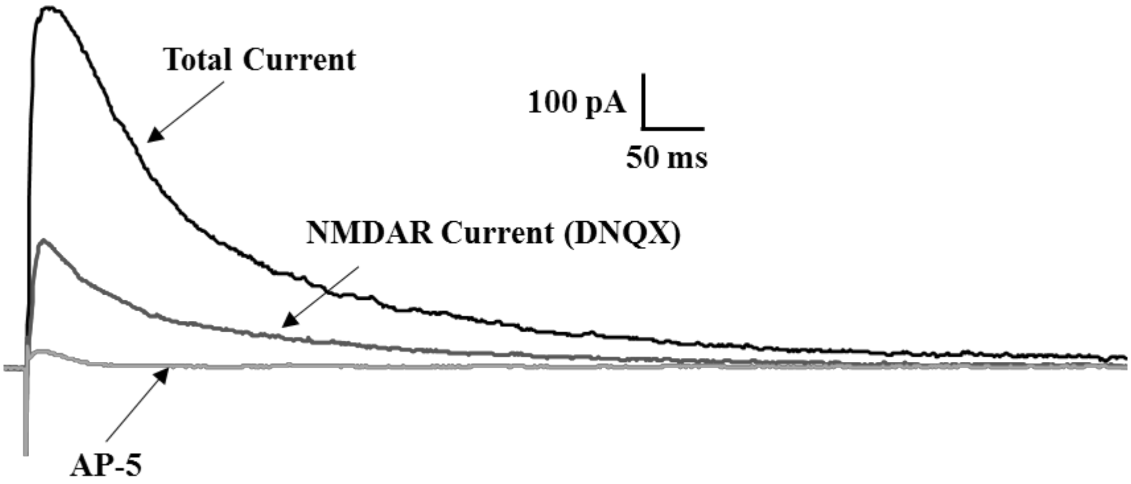

Supplementary Figure 1. Whole-cell patch clamp recording from CA1 hippocampal pyramidal neurons of aged and young animals demonstrating isolation of NMDAR EPSC.

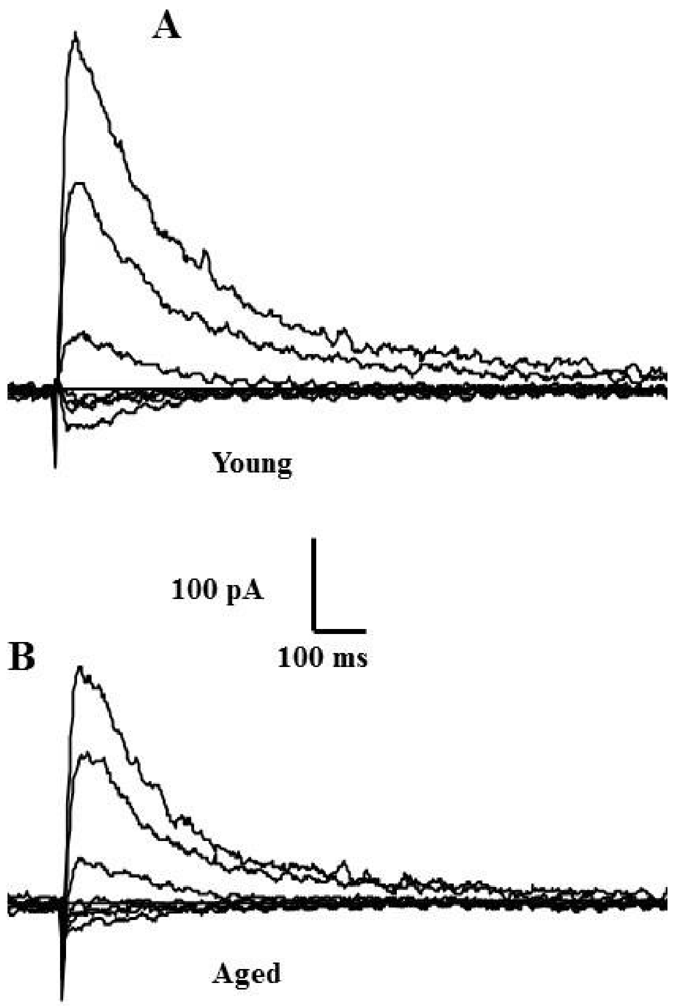

Supplementary Figure 2. Representative NMDAR EPSC traces recorded at a holding potential of -60, -40, -20, 0, +20, +40, and +60 mV from young **(A)** and aged **(B)** animals.
